# Molecular and structural imaging in surgically induced murine osteoarthritis

**DOI:** 10.1016/j.joca.2020.03.016

**Published:** 2020-07

**Authors:** N.H. Lim, C. Wen, T.L. Vincent

**Affiliations:** † Centre for Osteoarthritis Pathogenesis Versus Arthritis, Kennedy Institute of Rheumatology, University of Oxford, UK; ‡ Department of Biomedical Engineering, Hong Kong Polytechnic University, Hong Kong

**Keywords:** *In vivo* imaging, microCT, microMRI, Protease-activated probes, Optical imaging, Photoacoustic imaging, Osteoarthritis

## Abstract

Preclinical imaging in osteoarthritis is a rapidly growing area with three principal objectives: to provide rapid, sensitive tools to monitor the course of experimental OA longitudinally; to describe the temporal relationship between tissue-specific pathologies over the course of disease; and to use molecular probes to measure disease activity *in vivo*. Research in this area can be broadly divided into those techniques that monitor structural changes in tissues (microCT, microMRI, ultrasound) and those that detect molecular disease activity (positron emission tomography (PET), optical and optoacoustic imaging). The former techniques have largely evolved from experience in human joint imaging and have been refined for small animal use. Some of the latter tools, such as optical imaging, have been developed in preclinical models and may have translational benefit in the future for patient stratification and for monitoring disease progression and response to treatment. In this narrative review we describe these methodologies and discuss the benefits to animal research, understanding OA pathogenesis, and in the development of human biomarkers.

## Introduction

Our understanding of osteoarthritis (OA) pathogenesis has greatly increased since the advent of surgical models of OA in genetically modified mice^1^. An exponential increase in *in vivo* studies since this time has identified major molecular players in disease as well as excluding others^2^, ^3^, ^4^, ^5^, ^6^, ^7^. Whilst it is difficult to prove the clinical utility of murine models when there are so few successes in clinical practice, where they do exist, the results accord well. For instance, the lack of efficacy demonstrated by anti-cytokine therapy and the identification of nerve growth factor (NGF) as a target for OA pain holds true for both human and murine disease^7^, ^8^, ^9^, ^10^.

Pathological changes in mouse joints following surgical destabilisation are largely determined by histological assessment at multiple levels within the joint and mirror human disease well; demonstrating progressive cartilage degradation, osteophyte formation, modest synovial hypertrophy and late onset spontaneous pain behaviour. Although *in vivo* models were principally developed and validated to assess cartilage degradation as the main outcome measure, increasingly semi-quantitative assessments of bone and synovium are also being included in preclinical studies. The relative importance of each of these pathological features to symptomatic disease and cartilage loss is hotly debated in clinical and pre-clinical arenas. The ability to interrogate molecules in specific tissues of the joint by creating conditional knockout mice is likely to help elucidate these issues.

Animal imaging, both *in vivo* (live) and ex vivo (dead), offers adjunctive information that could be transformative in terms of screening genetically modified animals, understanding pathogenesis, and creating tools that could be useful for human disease monitoring. Specifically these include: (i) the development of rapid quantitative measures that circumvent the need for laborious histological processing and scoring in preclinical models; (ii) the development of prospective measures that could be used in live animals to follow the course of disease in an individual animal over time. This would have a significant impact on reduction of animals in research in line with ARRIVE guidelines; (iii) the ability to use disease activity probes that provide real time information on cellular processes associated with disease.

In addition to the benefits to the preclinical OA community, ultimately such imaging outcomes have clinical utility as biomarkers. The lack of sensitive biomarkers for OA greatly hampers progress in translation. The only widely accepted biomarker for OA is the Kellgren and Lawrence radiographic score, a composite of joint space narrowing and osteophyte formation, measured by plain X-ray. Joint space narrowing as a biomarker is insensitive, with only around 30% of individuals recruited to OA trials showing X-ray progression over a typical trial period, say 2–3 years^11^. Currently we are unable to predict which individuals progress and which stay stable over this period. The current lack of good biomarkers is a major limitation in clinical trial design and likely affects industry's decision to undertake drug development in this area.

Molecular imaging probes which report on particular disease activities could lead the way towards personalised medicine and stratification in human OA. For example, selective protease-activatable probes would be able to identify individuals who exhibit high levels of protease activity and monitor their response to protease-inhibitor treatments. Such activities may change over the course of disease and shift between different classes of proteases. One could envisage this type of molecular approach being applied to other selective therapies, for instance in stratifying patients more likely to respond to NGF neutralising or anti-inflammatory therapies.

In this review, we provide an overview of the currently available joint imaging modalities in mice, separating the review into those modalities that examine structural changes in joint tissues from those that examine molecular and disease activity changes within the joint. Whilst the focus is on murine joint imaging, we also describe methodologies in other rodents where we believe translation to mouse is realisable.

### Structural outcomes

Several imaging techniques have been deployed to study structural outcome measures in disease (Table I). Some of these, additionally can be used to generate functional and molecular information in a protocol specific manner (discussed below), or can be used in combination with molecular imaging to provide anatomical localisation. Generally, methodologies that are used for structural outcomes, provide higher resolution images than molecular imaging techniques (Table I). They are therefore good at providing sensitive and quantitative assessments of the joint. Increasingly these are being performed in live animals in which change can be followed over time in a single animal.

**Table I:** Comparison of pre-clinical imaging modalities

	μMRI	μCT	Ultrasound	PET	SPECT	Optical	Opto-acoustic
Imaging type	Structural	Structural	Structural	Molecular	Molecular	Molecular	Molecular
Resolution	150 μm/pixel	4.5 μm/pixel	30 μm/pixel	1 mm/pixel	250 μm/pixel	>1 mm/pixel	30 μm/pixel
Contrast agent	Gadolinium	Iodine	Microbubbles	^18^F	^99m^Tc	Fluorophore	Light-absorber
Typical amount of contrast required	mg	mg	mg	ng	ng	ng	ng-μg
Acquisition Time	>10 min to hours	<10 Mins	< secs	>10 min	>10 min	Secs	< sec

#### Computer tomography (CT)

**Fig. 1:**
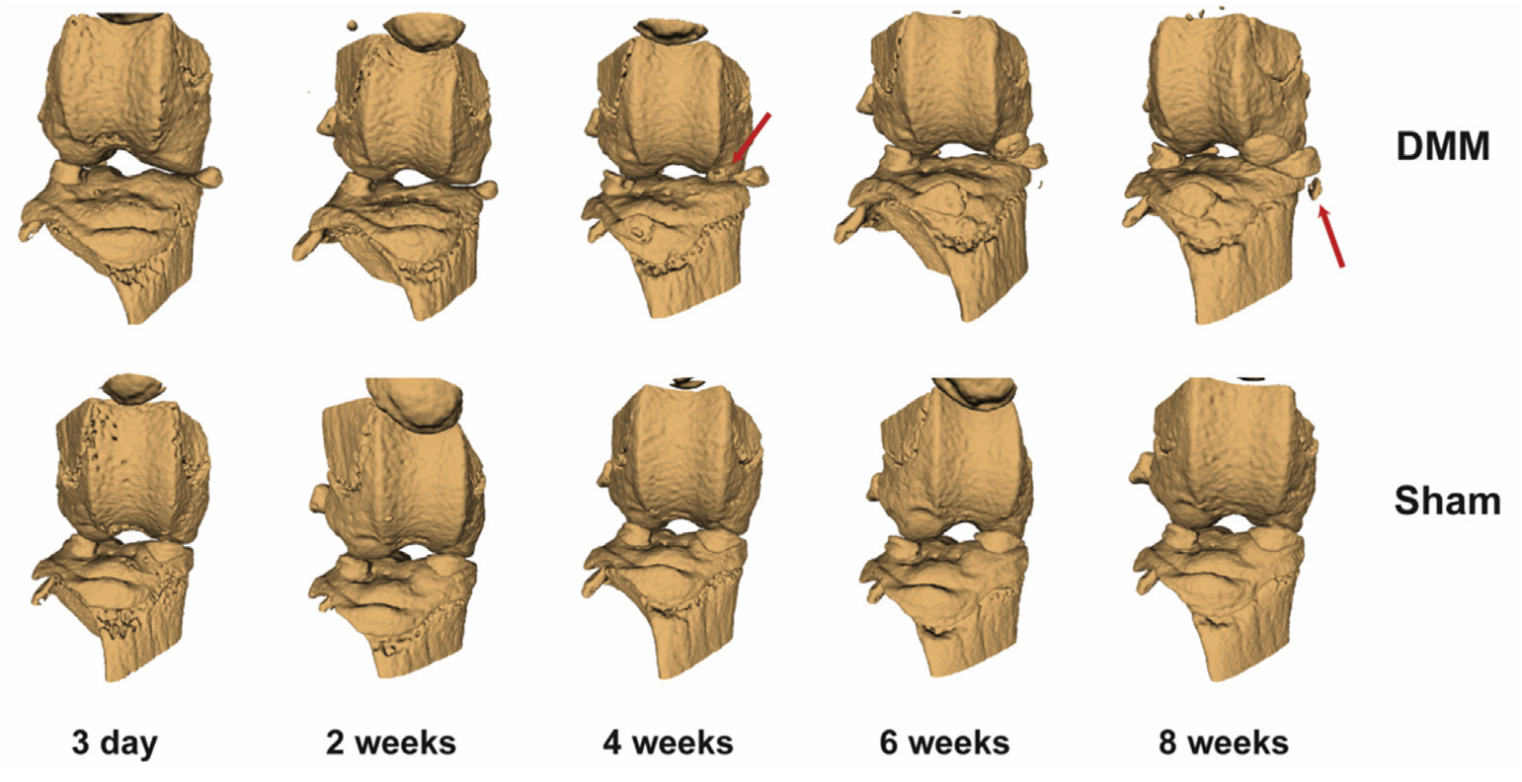
Prospective *in vivo* CT scans following changes within a joint after DMM or sham surgery (10 μm/pixel resolution). DMM surgery induces extrusion of the meniscus which calcifies in week 4 (red arrow). Ossification also occurs in the destabilised joint leading to formation of an osteophyte (week 8) (red arrow) (Lim, unpublished data).

Computer tomography (CT) is one of the most widely used *in vivo* and *ex vivo* imaging modalities in orthopaedic research. The mineral calcium in bone absorbs the X-rays and by rotating either the specimen or the X-ray source and detector, 3D data of the bone is reconstructed from multiple projections at multiple angles around the specimen^12^. With technological advancements in detector resolving power and computer processing power, high resolution *ex vivo* nanoCT scans of down to 50–600 nm per voxel are possible, allowing clear visualisation of minute features like osteocyte lacunae and vascular canals within a trabecula^13^. *In vivo* microCT scans are of lower resolution, in the 4.5–50 μm per voxel range, as it needs to balance motion artefact and the radiation dosage per scan. Oversampling with image binning overcomes motion artefact caused by heartbeats and respiration, but this increases the radiation exposure. *In vivo* CT allows the same animal to be followed throughout the study. Apart from reducing the number of animals required, this provides greater insight into the temporal development of structural disease, such as the increase in new bone (osteophytes) after surgical destabilisation of the medial meniscus (DMM) (Fig. 1).

**Fig. 2:**
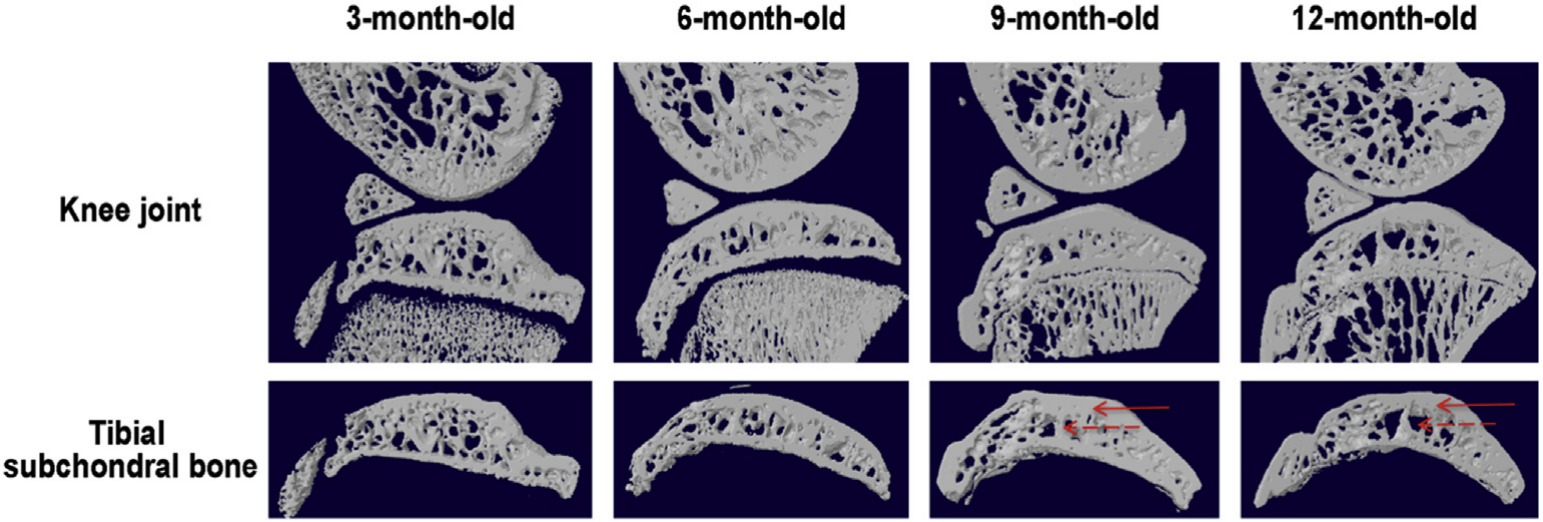
MicroCT changes in subchondral bone over time in spontaneous OA in the Hartley guinea pig. Note subchondral bone plate thickening and increased cystic lesion in underlying trabecular bone (Wen, unpublished data). Solid arrow – bone sclerosis; hashed arrow – cystic bone lesion.

Parameters that are typically assessed from CT scans in experimental OA include subchondral bone sclerosis and osteophyte size (Fig. 2). Osteophytes are defined as new bone formation and typically occur on the medial side of the joint following meniscal transection. Osteophytes are sometimes observed as small hook-shaped projections at the edge of the joint in CT scans, but they can also be inferred by an increase in volume of the epiphysis. Validation of osteophytes are performed by histology, in which new bone boundaries are clearly visible, and automated methods can be applied to speed up analysis^14^^,^^15^. Such quantitative volume assessments by microCT appear to map well to the development of the osteophyte by histology. Both subchondral bone thickening and osteophyte formation occur progressively after joint destabilisation. These are evident within the first couple of weeks of surgery by histology and epiphyseal volume measurements^14^. MicroCT has also been used to quantify osteophyte number, to describe bony deformity in genetically modified joints and abnormal ossification of soft tissues of the joint^16^, ^17^, ^18^, ^19^, ^20^, ^21^. Bone density measurements within the epiphysis are also measured although whether such features inform pathological processes in OA is unclear.

**Fig. 3:**
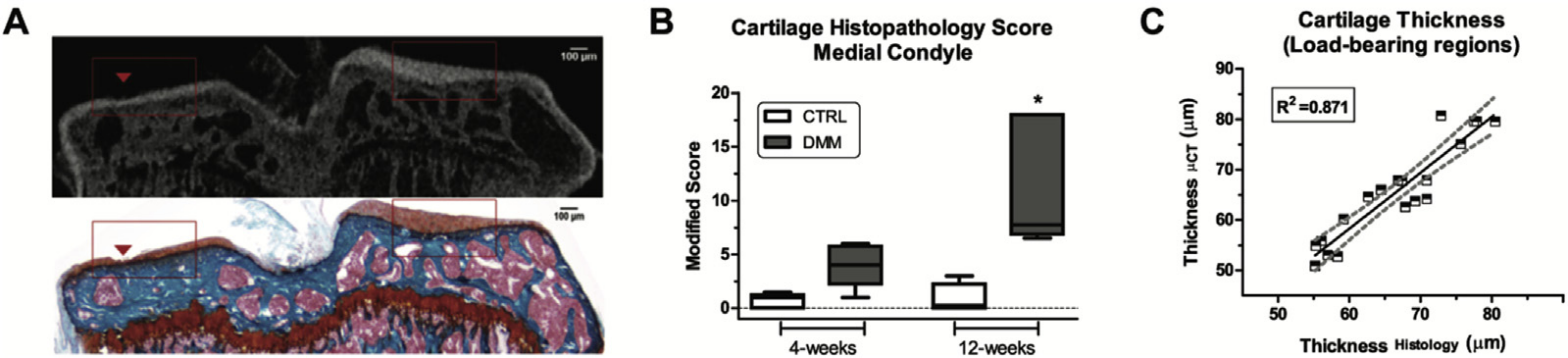
Quantitative cartilage volume assessment using *ex vivo* contrast enhanced microCT following DMM (A) *Ex vivo* tibia were stained with phosphotungstic acid and imaged by microCT and histology at 4 weeks post DMM (B) Histopathology scores of the medial condyles at 4 and 12 weeks post DMM (C) A good correlation is evident between the two cartilage thickness measurements using the two methods. Adapted from ^14^, with permission.

Cartilage is not visible by conventional CT unless a contrast agent is used. As with human contrast enhanced imaging, agents can either be anionic, thereby being excluded from GAG-rich matrix, or cationic, thereby being enhanced within cartilage. Both anionic and cationic contrast agents have been tested in rodent models of OA^14^^,^^22^, ^23^, ^24^. The correlation between cartilage damage assessment using this type of approach compared with histological scoring is excellent (^14^, adapted in Fig. 3). The different classes of contrast agents penetrate cartilage at different speeds and their *in vivo* utility can be broadened by adjusting the time of image acquisition after delivery, route of contrast agent delivery (intra-articular vs systemic) and dose (reviewed in^25^).

Contrast-enhanced nanoCT provides good spatial and contrast resolution equivalent to histological staining, and can provide fast and quantitative data on cartilage structure^13^. However, nano-CT is not yet suitable for live imaging due to extreme sensitivity to motion artefact. Synchrotron-radiation CT (SRCT), which generates high energy X-rays of narrower wavelength ranges, improves the resolution of conventional CT greatly. Intercortical pores in *ex vivo* murine bones are easily observed using SRCT^26^ and bones imaged by SRCT *in vivo* have better bone boundaries when imaged at a dose that is comparable to usual microCT, due to phase enhancement^27^. While conventional CT is poor at differentiating non-calcified tissues, Marenzana *et al.* demonstrated the use of SRCT for imaging mouse articular cartilage without the need for contrast agents^28^.

#### Magnetic resonance imaging

**Fig. 4:**
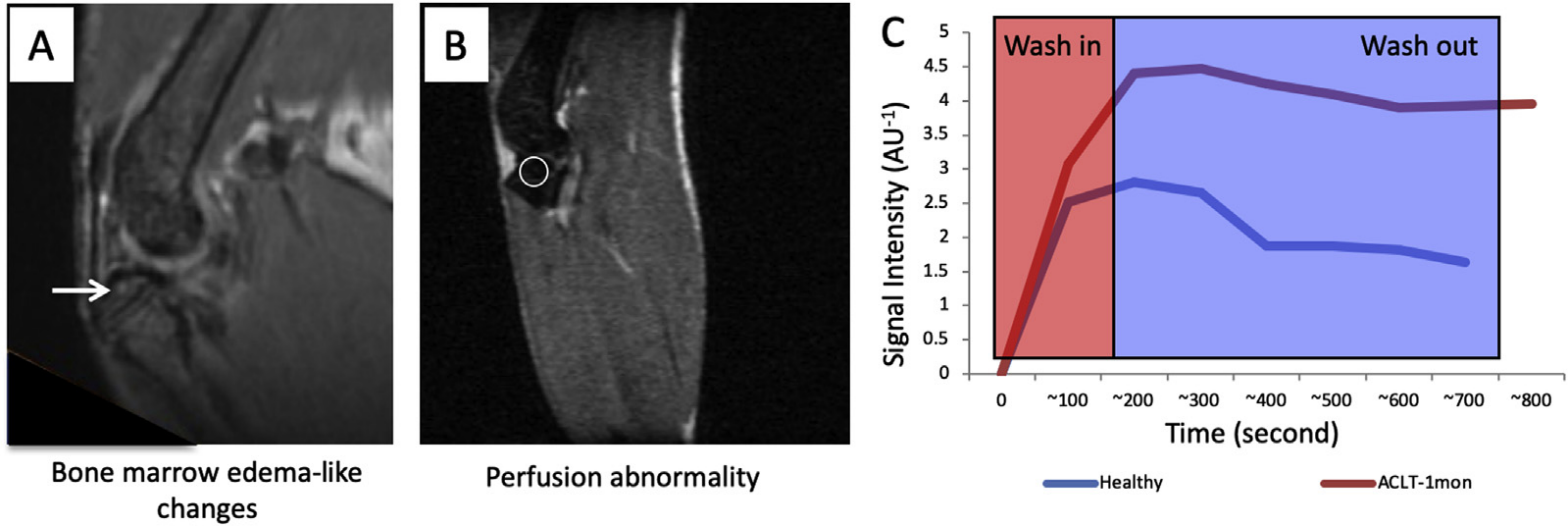
Perfusion abnormality in subchondral bone after transection of anterior cruciate ligament (ACLT) in C57BL/6 male mice (A) Subchondral edema-like changes (arrow) were found 1 month after injury in T2-weighted images of mouse knee joint using a horizontal 30-cm-bore 9.4T Bruker Biospec preclinical scanner (B) After injection of contrast agent - gadopentetate dimeglumine, T1-weighted MR imaging showed impaired blood perfusion with increased vascular permeability in femoral subchondral bone with decreases in bone marrow cavity. Little change was observed in muscle. Circle indicates where measurements were taken for subsequent quantification (C), adapted from ^29^, with permission.

Magnetic resonance imaging (MRI) utilises a strong electromagnet to generate a changing magnetic field at a frequency close to the natural frequency of hydrogen atoms. This allows hydrated tissues to be visualised well. Theoretically, MR imaging techniques allow optimal detection of several different joint tissues; from articular cartilage, meniscus, subchondral bone to synovium. Murine OA has been examined using a 9.4T preclinical micro-MRI scanner with an isotropic resolution of 0.068 mm/pixel for T2 images and 0.12 mm/pixel for T1 images^29^. However, the thickness of murine cartilage (80–100 μm) is close to the resolution limit of micro-MRI. Therefore, T2-weighted images are limited to detecting subchondral bone oedema-like changes and T1-weighted images to assessing perfusion abnormalities in subchondral bone after injection of contrast agent (Fig. 4). Subchondral bone edema and perfusion abnormalities were associated with increased angiogenesis which temporally preceded and spatially localised with bone and cartilage lesions^29^. Longitudinal micro-MRI imaging in surgically induced OA in rats showed that subchondral edema developed subsequently into cystic lesions^30^. Similar observations were also documented in spontaneous OA in the Dunkin Hartley guniea pig^31^.

The T2 relaxation time has also been derived to indicate structural integrity of articular cartilage in larger rodent models by probing free water proton movement inside the collagen-proteoglycan matrix. Indeed, T2 mapping has been used to detect proteoglycan loss and ECM alterations in rat patellar cartilage post hyaluronidase digestion^32^, and in spontaneous OA in the Dunkin Hartley guinea pig *in vivo*^33^. In a recent study by Ali *et al.*, pathological changes in the joint were assessed over time following rat meniscectomy. This study describes an initial swelling of the articular cartilage, subchondral bone remodelling, then proteoglycan depletion and cartilage erosion over 8 weeks^34^. Strategies that improve the contrast and specificity in MRI, such as by using sodium MRI or gadolinium (Gd), have been used in larger animals and may eventually have use in rodents^35^^,^^36^. Gd-containing cartilage targeting contrast agents, based on type II collagen binding, have been used to image cartilage in rats^37^.

#### Ultrasonography

**Fig. 5:**
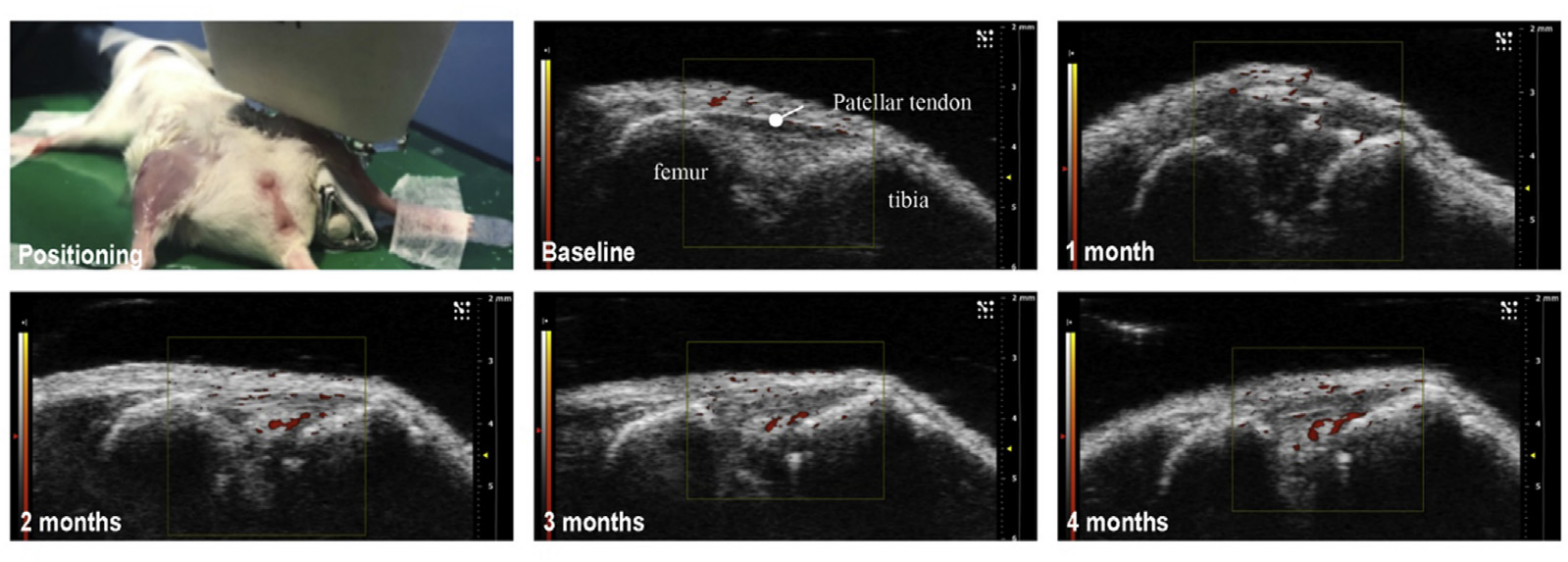
Ultrasound and Power Doppler imaging of intra-articular tissue damage and angiogenesis after destabilization of medial meniscus (DMM). Changes in intra-articular Doppler signals became evident by 2 months post DMM surgery (Wen, unpublished data). Similar findings are presented in^39^.

Ultrasonography has been used in clinical assessment of hand, hip and knee OA from grading synovitis, to measuring cartilage thickness, damage to the meniscus and assessing the cartilage–bone interface. In preclinical models, B-mode images have been used to assess synovitis in inflammatory arthritis^38^. Newer ultra-high frequency linear array transducers, such as MS700 with central frequency 30–70 MHz, have been developed with multi-focus capability to enhance tissue margin visualisation and provide improved morphological information such as synovial or meniscal swelling post injury^39^. Power Doppler can be applied to monitor joint blood flow over time, for example after destabilization of the meniscus where changes in vascularisation are evident (Fig. 5). Doppler imaging is reliant on moving objects in blood vessels, e.g., erythrocytes, and has a resolution down to 30  μm at an ultrasound probe frequency of 70 MHz. Cardiac pumping and respiratory rate of experimental mice under general anesthesia are major factors affecting power Doppler signals. Cardiac and respiratory gating can overcome this technical problem. B-mode ultrasound and Power Doppler are qualitative and often operator-dependent. Quantitative 3D Power Doppler ultrasonography has, to some extent, overcome operator-dependent observational bias and has been used to study posttraumatic murine osteoarthritis in a longitudinal follow-up study^39^^,^^40^. In this study, the authors determined joint space and blood flow volume of the joint. Interestingly, the change in blood flow volume determined by Power-Doppler was not apparent until 6-week post operation^40^. Ultrasonography has also been used *ex vivo* to study the joint surface (also termed biomicroscopy)^41^. Developing conjugated probes for arthroscopic use may provide future solutions^42^. Tissue resolution and penetration of ultrasonography currently limits its *in vivo* use; there being a trade-off between frequency of the ultrasound wave and penetration depth. Specifically it is unable to penetrate bone. Radiofrequency analysis of sonic signals at the bone-cartilage interface might be useful to delineate this functional unit during OA progression^43^.

### Molecular outcomes

Molecular imaging techniques detect selective molecular activities which can be monitored over the course of disease. Their value lies in the ability to explore pathogenetic mechanisms and validate therapeutic targets in real-time.

#### Positron emission tomograpy (PET)-CT

Positron Emission Tomography (PET) detects gamma rays emitted indirectly by positron-emitting radionuclide tracers. ^18^F-Fludeoxyglucose (FDG), which is the most commonly used positron emission tomography (PET) tracer, is taken up at higher rates at regions of heightened metabolic activity, e.g., cancer metastases. When used in combination with CT (PET-CT) skeletal localisation can be elucidated. Umemoto and colleagues examined rat ACLT-induced OA using ^18^F-fluoride PET and observed higher uptake of ^18^F in the operated knee starting 2 weeks post operation, particularly within the medial compartment^44^. Macrophage activity following induction of OA by mono-iodoacetate (MIA) was followed by PET using a ^64^Cu-peptide targeted to the formyl peptide receptor 1, which is up-regulated upon macrophage activation^45^.

#### SPECT

Single photon emission computer tomography (SPECT) is generated by use of a gamma-emitting radioisotope e.g., ^^99^m^technetium, ^123^iodine or ^131^iodine. Despite being less sensitive than PET, SPECT allows a longer time window for scans due to the longer half-life of tracers, thus allowing longitudinal imaging over days^46^. Real-time subchondral bone turnover during the onset and progression of MIA-induced OA was observed in rats using SPECT-CT imaging of a ^99m^Technetium-methylene diphosphonate radiotracer^47^. Activated macrophages were identified in the rat groove model of OA following SPECT imaging using a new DOTA-folate radio-conjugate^48^. A radio-tracer that was excluded from bone and synovium was used to image cartilage by SPECT^49^.

Improvements have been made to the pinhole design and camera design to improve resolution, magnification and detection efficiency^50^. The newest generation μSPECT system can resolve details down to 0.25 mm^51^. Furthermore, Hybrid SPECT/CT and SPECT/MRI systems have been designed and built for preclinical purposes to allow researchers to acquire functional images and structural images simultaneously, thereby understanding the correlations between metabolic activities and structural alterations.

#### Optical Imaging

Optical imaging detects light emitted in the visible to near infrared wavelengths. This light may either be from a bioluminescent source, or the longer emission wavelength of a fluorophore excited at a shorter wavelength of light (fluorescence). This makes it more accessible than the traditional PET/SPECT molecular imaging, as no radioactive material needs to be handled. The main drawbacks of optical imaging stem from the natural tendency of the tissues of the body to absorb and scatter light, leading to low spatial resolution and an imaging depth of about 1–1.5 cm.

#### Optical imaging - bioluminescence

Gene expression changes can be studied *in vivo* using reporter mouse lines containing a luciferase gene under the control of a promoter of interest. Whole body optical imaging systems are used to capture the light emitted following injection of the luciferin substrate. The expression of aggrecan^52^ and NFκB^53^ post-DMM surgery and NFκB post-MIA injection^54^ have been reported using the corresponding reporter mice^52^. The expression of NFκB demonstrated a correlation with pain^54^ and a phase of increased NFκB expression immediately following DMM or sham surgery but lasting longer in the DMM group^53^.

Luciferase reporters can also be used in transplanted cell tracking experiments using the same optical imaging systems to demonstrate the presence of exogenous cells in repairing tissues of OA rats following ACLT^55^^,^^56^ and persistence of transplanted senescent auricular chondrocytes when injected into mouse joints to cause OA like disease^57^.

#### Optical imaging – fluorescence

Whole body *in vivo* optical imaging systems can also track and quantify far red and near infrared fluorescence. Applications include far red expressing reporter genes or conjugation of the fluorophore to a variety of molecules including recombinant proteins, antibodies, peptides, nanoparticles and drugs. In our experience, the sensitivity of detection of far-red fluorescent protein when expressed by cells is about an order of magnitude less than luciferase.

The far-red probe Cy5.5 has been conjugated to an antibody selective for reactive-oxygen damaged type II collagen^58^. When delivered to mice following DMM surgery evidence of oxidative damage could be detected as early as 4 weeks post op, preceding histological changes^59^. Nanosomes encapsulating a near infrared dye and conjugated to an antibody that binds native type II collagen were retained by OA knees at a stage of modest superficial damage suggesting that this may be a sensitive marker of early disease^60^. Collagen hybridising peptides have been used to detect stretch induced damage to tendons through their binding to exposed collagen triple helices^61^. Although not yet applied to *in vivo* models, these would potentially make interesting probes to test in OA. Fluorescence imaging of aggrecan content in murine cartilage has been performed *ex vivo* using octaarginine-labelled with rhodamine, a methodology based on octaarginine's positive charge^62^.

#### Photoacoustic imaging

**Fig. 6:**
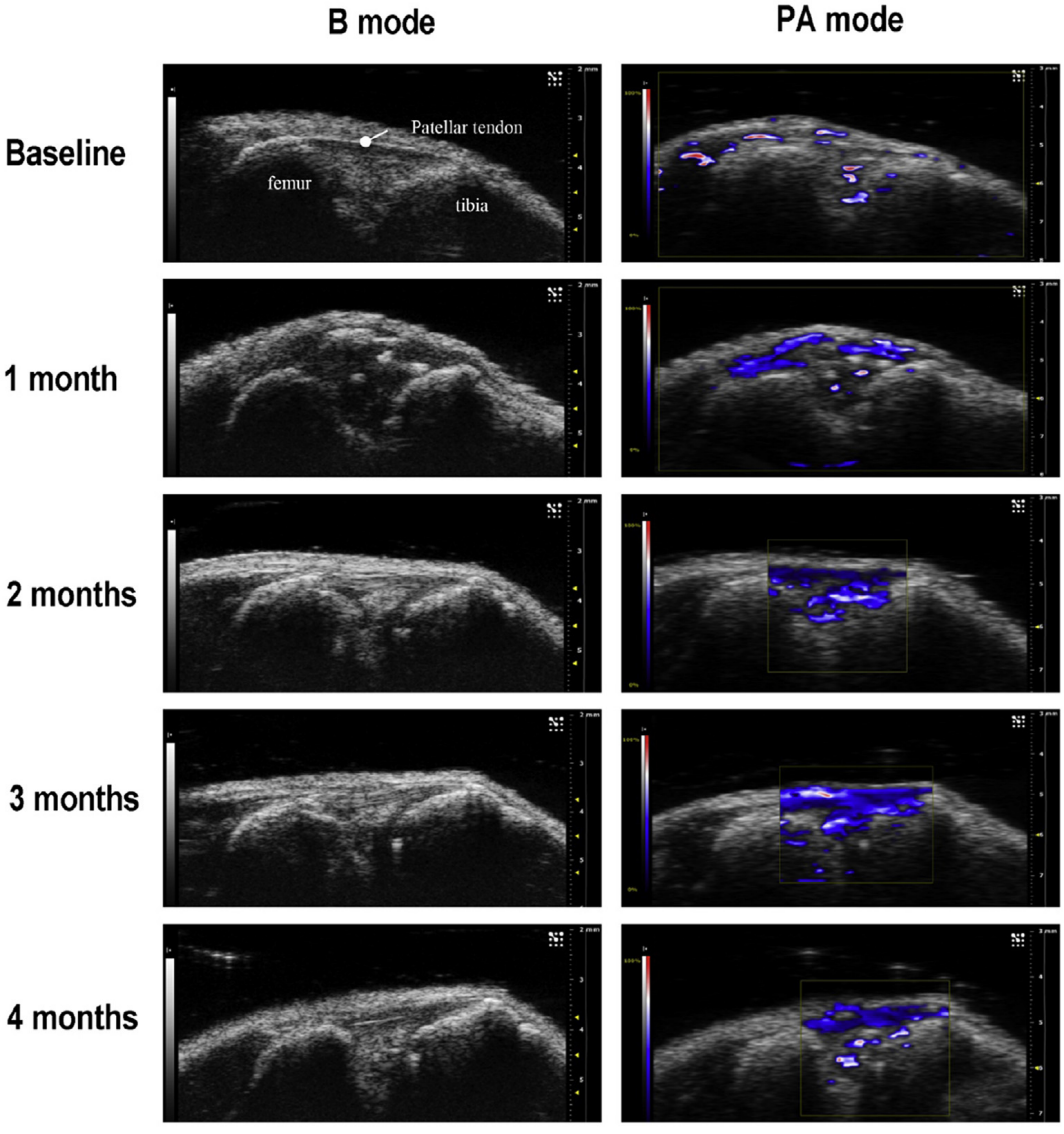
Ultrasound and photoacoustic imaging of intra-articular synovial and meniscal tissue angiogenesis in a DMM-induced posttraumatic osteoarthritis mouse model. Photoacoustic imaging reveals increased intra-articular tissue angiogenesis starting from 1 month post DMM surgery (Wen, unpulished data). Similar findings are presented in^39^.

Photoacoustic (PA) imaging, also known as optoacoustic imaging, is a derivative of ultrasonography. Unlike typical ultrasonography, no acoustic transducer is involved. Instead, a non-ionising laser source sends light to the tissues. This light energy is absorbed by the tissue and converted into heat, and it is this heat that drives rapid thermoelastic expansion, which in turns causes ultrasonic acoustic signal to be emitted. Emerging PA imaging exhibits strengths of both optical and sonic imaging, enabling one to probe the optical absorption properties of endogenous haemoglobin in blood vessels relatively deep in the tissue while achieving the spatial resolution of ultrasound. PA imaging can be applied to *in vivo* non-invasive measurements of tissue angiogenesis and oxygenation levels without contrast agent. Compared with Power Doppler, PA imaging appears more sensitive for *in vivo* detection of intra-articular tissue damage and angiogenesis. Following DMM in mice, a reduction in oxygen saturation and increase in vascularity correlated with cartilage damage over time^39^(Fig. 6). Changes in PA signal from the subchondral bone have been reported, albeit in a joint immobilisation model in rats^63^. A cartilage contrast agent for PA imaging, utilising melanine nanoparticles encapsulated in poly-l-lysine, has shown good correlation with tissue glycosaminoglycan (GAG) content and is able to distinguish early from late OA^64^.

Multispectral optoacoustic tomography (MSOT) is an advancement on PA and uses multiple wavelengths of light coupled with unmixing algorithms to excite both endogenous and exogenous light absorbing sources. MSOT, in combination with contrast, has been used successfully in inflammatory arthritis models and may have future application in OA imaging^65^.

#### Protease-activated probes

**Fig. 7:**
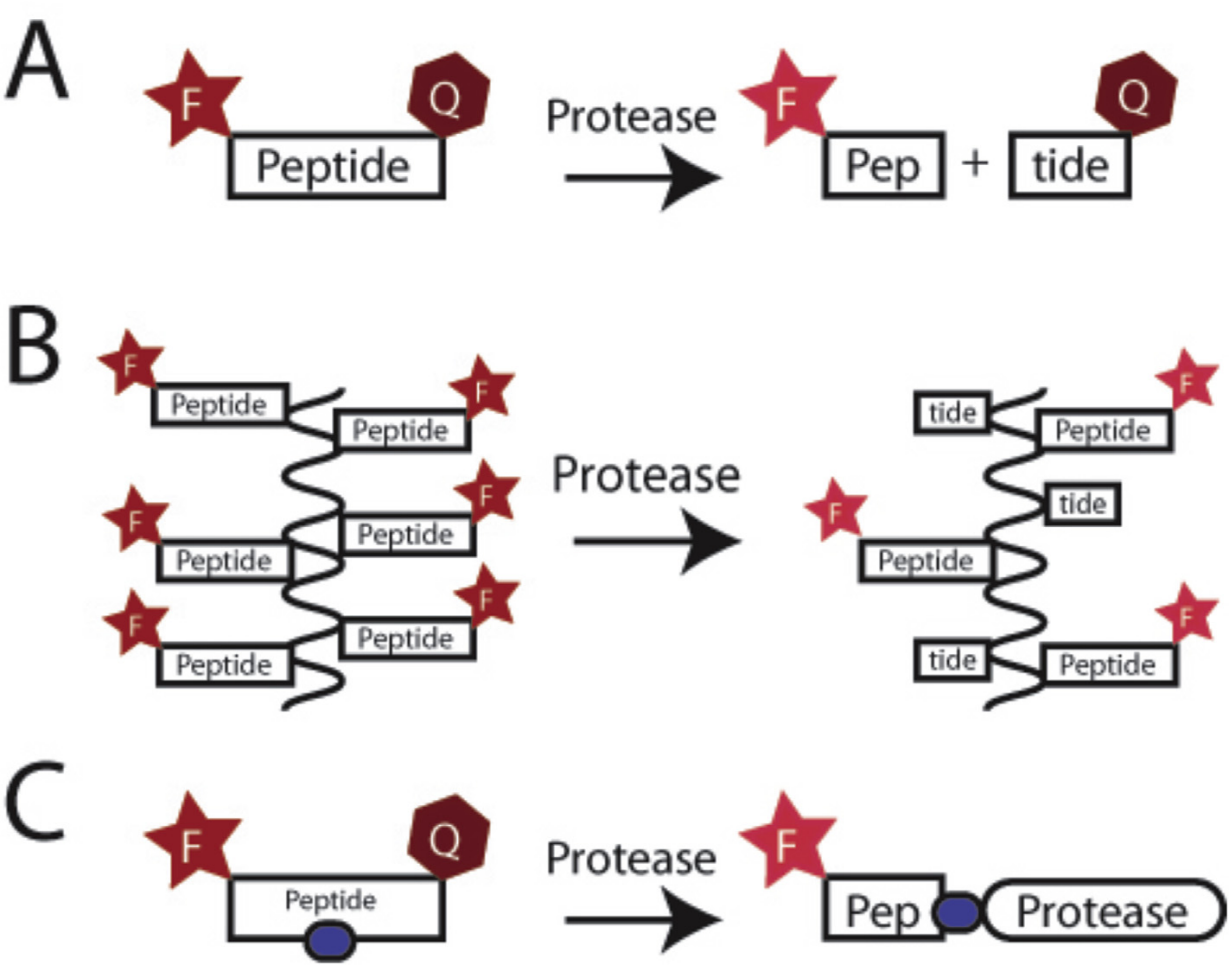
Schematic of protease specific probe activation. F-Star represents the fluorophore, Q-Hexagon the quencher and blue oval a chemical inhibitor moiety (A) A basic activity probe. In the intact probe, the fluorophore and quencher are in close proximity and any excitation of the fluorophore results in the transmission through fluorescence resonance energy transfer to the quencher. Upon cleavage, these separate and fluorescence is detectable (B) Activity probes attached to a polymer (e.g., MMPSense), without a chemical quencher. The high density of fluorophores results in self-quenching. Cleavage by protease releases some fluorophores and an increase in fluorescence (C) Activity probes with a hidden inhibitor moiety that will bind to the active site of the protease upon cleavage.

The ability to monitor specific protease activity *in vivo* in real time has huge potential both in terms of understanding the chronology of matrix degradation in OA but also as a biomarker for selecting or predicting response to a particular treatment. The probes work by utilising a cleavable peptide sequence, which only fluoresces upon cleavage (Fig. 7). The selectivity of the probe depends on the selectivity of the sequence of the peptide substrate. The absence of sensitive assays that would allow degradative processes to be measured quantitatively in OA tissues means that these types of approaches are especially valuable, but not so easy to validate.

Commercial activity based probes exist for the matrix metalloproteases matrix metalloproteinases (MMPs) and Cathepsins, with the brand names MMPSense and Prosense. Activity of these probes has been described in collagenase-induced OA as well as surgical destabilisation and mechanical injury models of OA. Unsurprisingly, highest signals have been obtained with the more inflammatory models and early in the post-operative period^21^^,^^66^, ^67^, ^68^. Cathepsin activity in particular appears to be more evident in inflammatory arthritis models such as collagen-induced arthritis rather than after DMM^69^. The Sensitivity of MMPSense680 activity is improved when the probe is delivered intra-articularly following DMM^70^. Prosense750 and MMPSense680 have also been used to monitor the efficacy of protease inhibition by the general protease inhibitor α2-macroglobulin and its more targeted variants^71^. These commercial probes, particularly MMPSense, were developed for cancer monitoring, and are not selective for particular MMPs, so activity cannot be assigned to a particular MMP without additional verification.

Probes with different specificities used in tandem may provide such validation. The different activation times of CatK680 compared with Prosense680 in the mechanical loading model suggests that Cathepsin K is activated early, whereas Cathepsin B, S, *L* or plasmin may be responsible for the later cleavage of Prosense680^67^. MMP13ap, which has moderately high selectivity for MMP-13 (also cleaves MMP-12), has been assessed after DMM in conjunction with MMP12ap (selective for MMP-12)^72^. As there was no regulation of MMP-12 observed with the MMP12ap probe, it is a reasonable assumption that the MMP13ap was indeed reporting on MMP-13 activity. Determining specificity may also be aided by parallel messenger ribonucleic acid (mRNA) analysis. For example, activation of MMPSense750 in the loading model may be due to MMP-3, as its mRNA was the upregulated, whereas MMP-9 and MMP-13 were unchanged^68^.

**Fig. 8:**
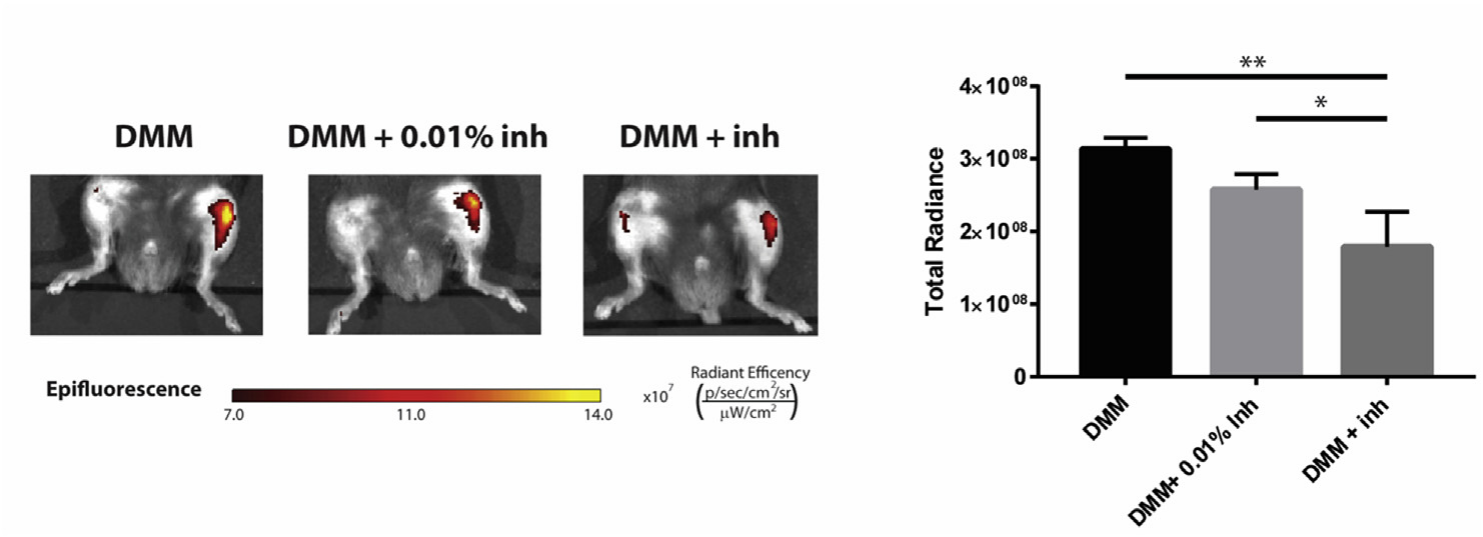
Optical Imaging of MMP-13 activity using PGA-P-18, showing inhibitor (A4727) efficacy at effective and non-effective (0.01%) doses, 8 weeks post DMM (Lim, unpublished data), in agreement with^76^.

Newer probes with increased specificity have been developed and it is beginning to be possible to elucidate the *in vivo* protease cascade during disease. The peptide sequence of the activity-based probe may be designed to increase the selectivity for a particular family member over the others. A peptide sequence, identified from a phage display library screen using MMP-13, was modified and used in an anterior cruciate ligament tear (ACLT) and menisectomy rat model and showed increased cleavage 6 and 8 weeks post-surgery^73^. A second generation probe showed reduced cleavage by MMP-2 and MMP-9, but was still cleaved by MMP-7^74^. Another method of obtaining selective peptide sequences is to reverse a selective peptide inhibitor into a substrate^75^, which was how MMP13ap was developed. The most recent reported MMP-13 probe (P-18) has used unnatural amino acids in order to achieve an almost 10-fold selectivity over the major confounding MMPs; MMP-2 and MMP-12^76^(Fig. 8). Coupled to a polyglutamic acid carrier (PGA-P-18), MMP-13 activity was detected 6 weeks post-DMM in mice and allowed the real-time readout of the efficacy of the MMP-13 inhibitor, A4727.

Development of probes for other proteases implicated in OA, such as the aggrecanases (ADAMTS-4 and ADAMTS-5), may provide earlier detection of OA and may allow stratification of targeted therapies.

## Conclusion

Radiographic biomarkers in preclinical disease have the ability to help unravel pathogenic processes relevant to human disease and in combination with *ex vivo* molecular approaches, define the role of each tissue over the course of disease. When measured prospectively, radiography has the ability to monitor disease progression in real time; reducing numbers of animals used and increasing the power of studies. Although contrast enhanced imaging of cartilage, either through microCT or photoacoustic imaging shows promise *in vitro*, these agents are not yet validated *in vivo* and the hope of finding a prospective imaging tool that will substitute for histological scoring of mouse joints is not currently within reach. Imaging probably holds more promise for those structural joint features that are less often reported; bone change, synovial vascularity and bone marrow oedema. Prospective imaging of bone remodelling after joint destabilisation is achievable by microCT scanning although caution should be taken not to over-interpret ossification of soft tissues, such as the meniscus, which occurs readily in mice as they age. Our experience is that epiphyseal volume change, the measurement of which can be automated, may be a more clinically relevant outcome measure^14^. Synovial hypertrophy and metabolic tissue status is not appreciated well by joint histology, so ultrasound technologies can add significantly to our understanding of the role of this tissue in OA. Limited resolution of microMRI continues to restrict its utility in non-inflammatory models of murine OA, although is the only imaging tool currently able to visualise bone marrow oedema.

Newer molecular probes are being validated in pre-clinical models and these offer significant potential; from charting the natural history of specific protease activities over the course of disease to validating new therapeutic interventions. Other aspects of disease pathogenesis, such as molecular determinants of pain could be explored using PET/SPECT, although current resolution will likely preclude precise tissue localisation *in vivo.* Ultimately, molecular imaging has important clinical translational promise in the search for early diagnostic, therapeutic and prognostic biomarkers.

## Contributions

TV and NHL conceived the review. NHL and CW collected data. All authors contributed to the writing of the manuscript and approved the final version.

## Conflict of interest

TLV and CW have no conflicts of interest to declare. NHL has a pending patent on diiodotyrosine containing contrast agents for use in cartilage imaging (WO2018020262A).
